# How should nonspecific complaints be defined? Comment to: “nonspecific complaints (NSCs) in the emergency department”

**DOI:** 10.1186/s13049-020-00805-x

**Published:** 2020-11-11

**Authors:** Roland Bingisser, Christian H. Nickel

**Affiliations:** Emergency Department, University Hospital Basel, University of Basel, Petersgraben 2, CH-4031 Basel, Switzerland

Dear Editor,

With great interest we have read the recent systematic review on “Nonspecific Complaints (NSCs) in the Emergency Department” [1] and would like to comment on two key aspects: First, we agree that NSCs should be considered a major emergency presentation. Different nomenclatures have been used in the past, such as “homecare impossible”, “unexplained symptoms”, “general disability”, “atypical symptoms”, and “nonspecific functional decline” (for review, see [2]). This diversity has not been helpful for a clinical definition or clinical research on nonspecific complaints [3]. Second, the authors did not present any definition of nonspecific complaints, but included studies using varying definitions from very different settings, such as Emergency Medical Services (EMS).

However, NSCs were first defined by Nemec et al [4] This original definition was based on the inclusion of patients in need of external resources, excluding patients with life-saving interventions. The original BANC studies excluded hemodynamically unstable patients (i.e. Emergency Severity Index (ESI) level 1; patients in need of life-saving interventions), as the work-up of shock is standardized. Similarly, the lowest triage categories were excluded as well, as these patients can usually be managed as see-and-treat outpatients and are not at risk for adverse outcomes. The rationale to focus on these patients is the lack of a standardized work-up, the high use of resources, and the risk of adverse outcomes [5–8]. All publications originating from the prospective multicenter Basel Nonspecific Complaints (BANC) cohorts used the above definition of NSCs [9–12], but other studies have used varying criteria to define NSCs [13]. There is an inherent difference regarding inclusion criteria between the studies considered by the present systematic review, e.g., some of the included studies were retrospective, and one study [14] has used a post-hoc classification of nonspecific complaints [15].

Surprisingly, the original publication by Nemec et al. [4], coining the term, was excluded from the present systematic review. We believe that this was based on arbitrary inclusion criteria. The argument to exclude studies deliberately focusing on certain triage categories does not seem valid unless an alternative, stringent definition of NSCs is used.

Therefore, we suggest that further research should be based on a common definition of nonspecific complaints in emergency presentations, in order to reduce heterogeneity of studies.

## Data Availability

NA

## Abbreviations

NSCs: Nonspecific complaints
BANC study: Basel nonspecific complaints study
ESI: Emergency severity index
